# Polymorphism T81C in H-RAS Oncogene Is Associated With Disease Progression in Imatinib (TKI) Treated Chronic Myeloid Leukemia Patients

**DOI:** 10.14740/wjon912e

**Published:** 2015-04-12

**Authors:** Rashid Mir, Imtiyaz Ah, Jamsheed Javid, Mariyam Zuberi, Sameer Guru, Masroor Mirza, Shazia Farooq, Prasant Yadav, Prakash C. Ray, Naresh Gupta, Alpana Saxena

**Affiliations:** a Faculty of Applied Medical Sciences, University of Tabuk, Tabuk 71491, Saudi Arabia; b Cancer Genetics Lab, Department of Biochemistry and Associated Hospitals, New Delhi, India; c Department of Medicine, Maulana Azad Medical College and Associated Hospitals, New Delhi, India; d These authors contributed equally to this paper.

**Keywords:** H-RAS T81C polymorphism, Restriction fragmentation length polymorphism, H-RAS, K-RAS, N-RAS, Chronic myeloid leukemia

## Abstract

**Background:**

Mammalian cells contain three functional RAS proto-oncogenes, known as H-RAS, K-RAS, and N-RAS, which encode small GTP-binding proteins in terms of p21^ras^s. RAS genes have been elucidated as major participants in the development and progression of cancer. A single nucleotide polymorphism (SNP) at H-RAS cDNA position 81 T→C (rs12628) has been found to be associated with the risk of many human cancers like gastrointestinal, oral, colon, bladder and thyroid carcinomas. Therefore, we hypothesized that this polymorphisms in H-RAS could influence susceptibility to chronic myeloid leukemia as well, and we conducted this study to test the hypothesis in Indian population.

**Method:**

H-RAS polymorphism was studied in 100 chronic myeloid leukemia (CML) patients and 100 healthy controls by restriction fragmentation length polymorphism (RFLP-PCR). Associations between polymorphism and clinicopathological features of CML patients were investigated.

**Results:**

In CML patients, the TT, TC and CC genotype frequency was 38%, 61% and 1% respectively, compared to 92%, 8% and 0% in healthy controls respectively. Compared to TT genotype, CT was significantly associated with increased risk of CML (odds ratio (OR): 8.4, P < 0.00001). There was a statistically significant correlation of H-RAS polymorphism with phases (P < 0.0003), molecular response (P < 0.0001), hematological response (P < 0.04) and thrombocytopenia (P < 0.003). However, there was no correlation of this polymorphism found with other clinical parameters.

**Conclusion:**

H-RAS T81C polymorphism was found to be associated with CML risk and prognosis of CML. These results suggest that C heterozygosis may be considered a potential risk factor for CML development in the North Indian population.

## Introduction

Chronic myeloid leukemia (CML) is a clonal myeloid stem cell disorder characterized by excessive accumulation of clonal myeloid cells in hematopoietic tissues. Clinically CML can be divided into three phases: the chronic phase (CP), the accelerated phase (AP), and the blast crisis (BC) [1, 2]. It occurs with an annual incidence of 1.0 - 1.5 per 100,000 persons [3]. CML occurs very rarely in children. More than 90% of CML patients are diagnosed when their disease is in a relatively early phase known as the CP [3]. CML is characterized by the presence of the Philadelphia (Ph) chromosome, a shortened 22 chromosome resulting from a (9;22) (q34;q11) reciprocal translocation that juxtaposes the c-abl oncogene 1 (ABL1) gene on chromosome 9 with the breakpoint cluster region (BCR) gene on chromosome 22 [4]. This reciprocal translocation gives rise to BCR-ABL fusion oncogene, resulting in fusion messenger RNA molecules (e1a2, b2a2, b3a2, and e19a2) of different lengths that are translated into different chimeric protein products (p190, p210 and p230 BCR-ABL respectively) that are characterized by constitutive activation of its tyrosine kinase activity [5]. H-RAS, K-RAS, and N-RAS are the three functional RAS proto-oncogenes, which encode small GTP-binding proteins. RAS genes have been characterized as major participants in the development and progression of a series of human tumors, such as gastrointestinal cancer, lung cancer, thyroid cancer, melanoma, and breast cancer [6]. H-RAS, N-RAS, and K-RAS proteins are widely expressed, with K-RAS expressing in almost all cell types. Members of the RAS gene family code for proteins of molecular weight 21,000 (p21); these proteins are membrane bound. They bind with GTP during their active state and GDP during their inactive state. The switch between their inactive and active forms, together with their ability to bind to target proteins, provides the mechanism for the cell differentiation, development and proliferation [7, 8]. Harvey-RAS (H-RAS) gene is located on chromosome 11 [9]. Oncogenic point mutations, namely the hotspot mutations in codons 12, 13 and 61, are frequently observed in many tumor types [10]. Besides the mutation hotspots, inherited polymorphisms in the H-RAS sequence were described [11, 12]. A single nucleotide polymorphism (SNP) at H-RAS cDNA position 81T/C (rs12628), in codon 27 of exon 1, was shown to be associated with the risk of human cancers [13]. H-RAS T/C polymorphism does not impair p21 protien structure and function as both CAT and CAC codons encode histidine (His27His). However, it was recently demonstrated that the C allele of this SNP could increase the risk of urinary bladder cancer, colon, and gastric cancer [14-16].

Therefore, in the present research, we hypothesize that the H-RAS T81C polymorphism may have an effect on the H-RAS activity, and ultimately may play a role in modulating the susceptibility to leukemia. In order to verify our hypothesis, a population-based case-control study was conducted to investigate the association between the H-RAS T81C genotypes and the risk of CML in Indian population.

## Material and Methods

### Sample collection

Peripheral blood samples, i.e. 5 mL venous blood samples, were collected in EDTA vials from 100 CML patients as well as from 100 healthy donors. Buffy coat was isolated and washed in red cells lysis buffer. All samples were stored at -80 °C until the RNA and genomic DNA was extracted. The diagnosis was confirmed by detecting t(9;22) or BCR/ABL fusion gene (p210^bcr-abl^) which is further categorized into b3a2 or b2a2 subtypes on the basis of the BCR breakpoint by reverse transcription polymerase chain reaction in the Molecular Oncology Lab, Department of Biochemistry.

### Selection criteria of patients

#### Inclusion criteria

The study included newly diagnosed CML patients treated with imatinib mesylate with a dose of 400 to 800 mg/day. All three stages of cases were included: CP, AP, and BC.

#### Exclusion criteria

The exclusion criteria included chronic myelomonocytic leukemia (CMML) patients, and other myeloproliferative disorder patients. The patients’ follow-up was maintained regularly and samples were collected after every 6 months for imatinib response and mutation studies. The classic criteria used for imatinib mesylate responses in CML for hematologic and molecular responses are depicted in Tables 1 and 2.

**Table 1:** Hematologic Responses

Complete or major hematological response	Partial or minor hematological response	Lose or minimal hematological response
Platelet count >150 × 10^9^/L WBC count < 10 × 10^9^/L Basophils: < 5% Differential without immature granulocytes Absence of blasts and promyelocytes in peripheral blood Spleen: non-palpable spleen	Platelet count < 450 × 10^9^/L WBC count > 10 × 10^9^/L Basophils: > 10% Presence of blasts and promyelocytes in peripheral blood Spleen: palpable spleen	Platelet count < 450 × 10^9^/L WBC count > 20 × 10^9^/L Basophils: 15% Presence of blasts and promyelocytes in peripheral blood Spleen: palpable spleen

**Table 2:** Molecular Response

Major molecular response	Minimal or no molecular response
It indicates non-quantifiable and non-detectable BCR-ABL gene transcript (BCR-ABL/ABL) ≤ 0.10^3^* check every 3 months	It indicates quantifiable and detectable BCR-ABL gene transcript (BCR-ABL/ABL) ≥ 0.10^3^* check every 3 months

*BCR-ABL to control gene ratio according to international scale (IS).

### RNA isolation

Total RNA was isolated from mononuclear cells with guanidinium isothiocyanate (Trizol LS^TM^, Invitrogen), according to the protocol provided by the manufacturer. The presence of RNA was confirmed by running the product on 2% agarose gel.

### cDNA synthesis

The concentration of RNA was measured spectrophotometrically. cDNA was then synthesized using M-MuLV reverse transcriptase and other reaction components (Fermentas Cat. No. K1622), according to the protocol provided by the manufacturer.

### Multiplex RT-PCR for BCR-ABL

BCR-ABL transcripts were detected using allele-specific primers for p210 and p190 primer sequences, as already described [17] listed in Table 3. PCR was carried out in a total volume of 25 μL reaction mixture containing 1 U/μL Taq polymerase, 240 μM dNTP, 1.8 M MgCl_2_, and 0.6 μM of primers. A program was employed, under the following conditions: an initial denaturation step at 95 °C for 10 min, then followed by 40 cycles of denaturing at 94 °C for 40 s, primer annealing at 55 °C, extension at 72 °C for 45 s, and a final extension step at 72 °C for 5 min. The expected bands were as follows: 808 bp, normal BCR; 481 bp, e1a2; 385 bp, b3a2; 310 bp, b2a2.

**Table 3:** Sequence of Oligonucleotides Used in Multiplex RT-PCR for Detection of BCR-ABL Transcript as the Target Gene and BCR Transcripts as the Internal Control

BCR-ABL primers
C5e 5'-ATAGGATCCTTTGCAACCGGGTCTGAA-3'
B2B 5'-ACAGAATTCCGCTGACCATCAATAAG-3'
BCR-C 5'-ACCGCATGTTCCGGGACAAAAG-3'
CA3 5'-TGTTGACTGGCGTGATGTAGTTGCTTGG-3'

### DNA extraction

Genomic DNA from peripheral blood was extracted using genomic DNA extraction kit (Gene Aid Cat. No. GB 100). The quality and integrity of the DNA was determined by the A260/280 ratios.

### H-RAS T81C genotyping

H-RAS T81C polymorphism located in exon 1 was analyzed by RFLP-PCR using DRAIII enzyme. Restriction fragmentation length polymorphism (RFLP) assay was performed at cancer genetics laboratory, MAMC, New Delhi. A 200-bp segment was amplified by RFLP primers reported from elsewhere [14] represented in Table 4.

**Table 4:** Sequence of Oligonucleotides Used in Allele Specific PCR of H-RAS Gene

Gene	H-RAS
Forward primer	5'-CTTGGCAGGTGGGGCAGGAGA-3'
Reverse primer	5'-GGCACCTGGACGGCGGCGCTAG-3'

**Figure 1:**
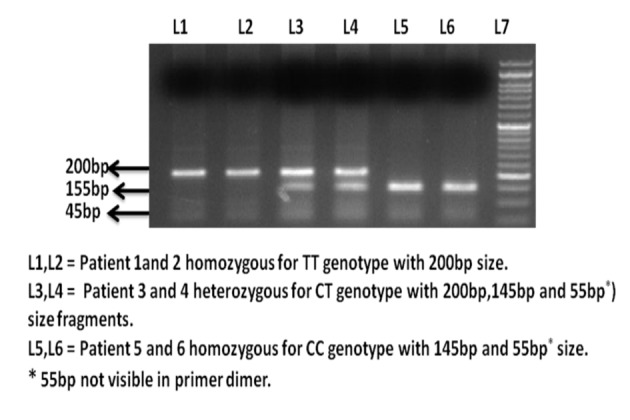
Ethidium bromide stained agarose gel electrophoresis image of H-RAS T81C polymorphism.

The amplification was accomplished with a 25 μL reaction mixture containing 5 μL of 20 ng template DNA, 0.25 μL of 25 pmol each primer, 2.5 μL 10 mM dNTPs, 1.5 μL of 20 mM MgCl_2_, 0.3 μL of 5 U/μL Taq polymerase with 2.5 μL of 10× Taq buffer (FD1224, Fermantas). The amplification conditions were 10 min of initial denaturation at 95 °C, 40 cycles at 95 °C for 45 s, annealing temperature at 60 °C for 45 s and 72 °C for 45 s with a final 10 min extension step at 72 °C. Amplified PCR product (15 μL) was digested with 1 μL of 10 × digestion buffer containing 3 units fast digestion DraIII ((FD1224, Fermantas). After 15 min digestion at 37 °C, the fragments were separated on a 2% agarose gel stained with ethidium bromide. TT homozygote presented a single fragment of 200 bp, the CC homozygote with DraIII restriction site was cut into fragments of 145 bp and 55 bp, whereas the heterozygous CT genotype presented all the three fragments as shown in Figure 1.

### Statistical analysis

CML patients and controls were compared by statistical analysis performed using the SPSS 16.0 software package. Chi-square analysis and Fisher exact test were carried out to compare H-RAS T81C frequency with several clinical aspects, including sex, age, stage, BCR-ABL type, and thrombocytopenia. Hardy-Weinberg equilibrium was tested by a χ^2^ test to compare the observed genotype frequencies within the case-control groups. The associations between H-RAS T81C variant genotypes and risk of CML were estimated by computing the odds ratios (ORs) and their 95% confidence intervals (CIs) from both univariate and multivariate logistic regression analysis. P value of ≤ 0.05 was considered statistically significant.

## Results

The study includes 100 CML patients and 100 healthy controls frequency matched with age and gender. A total of 65 males and 35 females already diagnosed with CML were included in the study. Two age groups were made, patients aged > 45 years included 36 cases, and above < 45 included 64 cases. The demographic characteristics of the study subjects, age and gender of patients, BCR-ABL type, thrombocytopenia, molecular response and hematological response are summarized in Table 5. Patients with a history of previous cancer were excluded. The Institutional Ethics Committees of Maulana Azad Medical College, New Delhi, India, approved the study and all patients provided written informed consent.

**Table 5:** Clinical-Pathological Parameters of CML Patients

	No.	%
Patients	100	100%
controls	100	100%
Males	65	65%
Female	35	35%
CP-CML	50	50%
AP-CML	25	25%
BC-CML	25	25%
Imatinib	100	100%
MMR	52	52%
No MR	48	48%
MHR	50	50%
Minor HR	10	10%
Loss HR	40	40%
Age > 45	36	36%
Age < 45	64	64%
Thrombocytopenia	50	50%
No thrombocytopenia	50	50%

### H-RAS T81C genotyping frequency

We identified frequent T>C variation in codon 27 of exon 1 at cDNA position 81 of H-RAS, and the frequency of H-RAS genotypes for TT, CT and CC was 38%, 61% and 1% in patients compared to 92%, 8% and 0% in healthy controls. A statistically significant difference was observed between patients and healthy controls (P > 0.0001). Genotyping results are summarized in Table 6. The frequencies of T allele and C allele were 0.69 and 0.32 and allele frequencies were consistent with Hardy-Weinberg equilibrium. The frequency of C rare allele observed in CML was about 32.2% which was significantly higher than that in controls 4.1% (χ^2^ = 48.7, P < 0.00000001). However, there was a non-significant correlation found between H-RAS T81C polymorphism and the clinical features like age, gender, imatinib therapy and BCR-ABL transcripts.

**Table 6:** Association of H-RAS T81C Polymorphism With the Clinicopathological Features

Clinical features	No.	TT	CT	CC	P value
Patients	100	38	61	1	< 0.0001
Controls	100	92	8	0	
Males	65	25	39	1	< 0.7
Female	35	13	22	0	
CP-CML	50	29	20	1	< 0.0003
AP-CML	25	7	18	0	
BC-CML	25	2	23	0	
A2b2	31	13	18	0	< 0.9
A2b3	67	24	42	1	
A2b2/A2b3	2	1	1	0	
Imatinib	100	38	61	1	
MMR	52	31	21	0	< 0.0001
No MR	48	7	40	1	
MHR	50	26	24	0	< 0.04
Minor HR	10	3	7	0	
Loss HR	40	9	30	1	
Age > 45	36	11	24	1	< 0.2
Age ≤ 45	64	27	37	0	
Thrombocytopenia	50	11	38	1	< 0.003
No thrombocytopenia	50	27	23	0	

### H-RAS genotype frequency in CML phases

**Figure 2:**
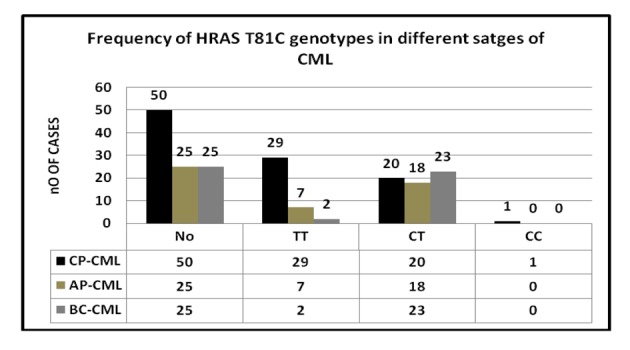
Association of H-RAS T81C with stages of CML.

A statistically significant correlation was found between H-RAS genotypes in CML phases. The rare C allele considerably increased from chronic phase to advanced phase patients. The genotype frequencies for TT, CT and CC were 58%, 40% and 2% in CP, 28%,72% and 0% in AP and 8% ,92% and 0% in BC respectively (Fig. 2). The CT heterozygosity was a common feature of AP and BC phase CML compared to TT homozygote suggesting that H-RAS T81C polymorphism plays a significant role in the progression of CML.

### Correlation of H-RAS polymorphism with molecular response

Among the 100 CML patients at the time of analysis, 52 displayed major MR, 48 minimal or no molecular responses. In MMR (major) group, 52.6%, 47.4% and 0% patients displayed TT, CT and CC genotypes compared to 17.5%, 83.3% and 0.2% genotypes in m HR (minor) respectively. A statistically significant difference was found between the two groups (P < 0.0001).

### Correlation of H-RAS T81C polymorphism with hematological response

Fifty CML patients had major HR, 10 minor HR and 40 minimal hematological responses respectively. The frequencies of H-RAS T81C genotypes TT, CT and CC were 52%, 48% and 0% in MHR, 30%, 70% and 0% in minor HR and 22.5%, 75% and 2.5% in loss HR group respectively. A significant correlation (P < 0.04) was reported between H-RAS polymorphism and hematological response in CML patients.

### Correlation of H-RAS T81C polymorphism with thrombocytopenia

Higher percentage of heterozygosity was detected in patients with thrombocytopenia. The genotype frequencies were 22%, 76% and 2% in thromocytopenia group and 54%, 46% and 0% in no thrombocytopenia group for TT, CT and CC genotypes respectively. A statistically significant correlation was detected (P = 0.003).

### H-RAS T81C polymorphism and CML risk

An unconditional logistic regression was used to estimate associations between the genotypes and risk of CML (Table 7). It was found that an increased risk of CML was associated with the C allele. Compared to the TT genotype, the OR of 18.4 (8.0 - 14.2) for the CT genotype, it was found that there was about more than 18-fold risk associated with heterozygosity, suggesting a possible dominant effect of this polymorphism in CML Indian population. Thus, carrying out OR calculation, it became possible to assess whether the occurrence of the polymorphic variants of H-RAS gene at codon 27 was in any way associated with the increased susceptibility to CML.

**Table 7:** Risk of CML Associated With the H-RAS Genotypes

Genotyping	Cases (n = 100)	Control (100)	OR* (95% CI)	P value
TT	38 (38%)	92(92%)	1	
TC	61 (61%)	8 (8%)	18.4 (8.0-14.2)	< 0.0001
CC	1 (61%)	0 (0%)	-	
Allele type				
T allele	136(68.6%)	184(95.8%)	1	
C allele	62 (32.2%)	8 (4.1%)	10.4 (4.8-22.6)	< 0.0001

OR: odds ratio.

## Discussion

RAS small GTPases are activated in many hematopoietic growth factor signaling and in hematological malignancies, but their role in hematopoiesis and leukemogenesis is not completely known. In addition to their normal cellular functions, RAS proteins play critical roles in tumorigenesis. Several molecular alterations in the RAS genes have been identified such as minisatellites and acquired mutations in various cancers. Mutated RAS genes are associated with approximately 30% of all human cancers, including both solid tumors and hematological malignancies [10].

In addition to the direct activation by mutations, RAS can also be functionally activated by other oncogenic mutations, such as the BCR/ABL fusion protein. Likewise, increased levels of RAS activation have been linked to CML resulting from the Brc/Abl translocation creating the Philadelphia chromosome [10]. Mutations in the RAS family of proto-oncogenes (H-RAS, N-RAS, and K-RAS) appear frequently in acute leukemias [18, 19]. Moreover, mutations in RAS genes may on occasion contribute to the transition from chronic-phase CML to the BC [20].

The role of H-RAS gene polymorphism in the pathogenesis of CML has not been reported. It has been suggested that H-RAS gene acts as enhancers of gene transcription. It was reported that polymorphic loci SNP 81T>C can indicate an increased risk of different types of human tumors, bladder, oral, gastrointestinal cancers, breast, lung, and skin [14, 16, 21-24]. H-RAS 81T-C polymorphism may induce aneuploidy through overexpression of the active p21 isoform of H-RAS in follicular thyroid tumors [25]. SNP 81T3C, although it does not alter the amino acid sequence of the p21 RAS protein, may affect the expression of the gene inducing overexpression [26]. It is conceivable that SNP 81T>C is linked to other polymorphic sites in functional intron regions of H-RAS.

We identified frequent polymorphism in the coding region of H-RAS in cDNA position 81T>C, which appeared to be a marker for the risk of CML development and progression. Our results show that heterozygote carriers of the variant C allele at the site of C/T polymorphism at codon 27 of the H-RAS gene have a decreased risk of CML. To confirm this, we screened cases and controls for T81C polymorphism. An unconditional logistic regression was performed to find the association of H-RAS polymorphism with CML; we found an 18-fold increased risk of CML in carriers of the heterozygous CT genotype in comparison to wild-type carriers (TT) (P > 0.0001, OR: 18.4). H-RAS 81 C allele has been shown to be a dominant genetic susceptibility factor for the development of many cancers in comparison to T allele; individuals harboring the homozygous and heterozygous 81C-genotype are at an increased risk of thyroid cancer, gastric cancer, colon cancer, bladder and oral cancer [14-16, 27-29].

**Figure 3:**
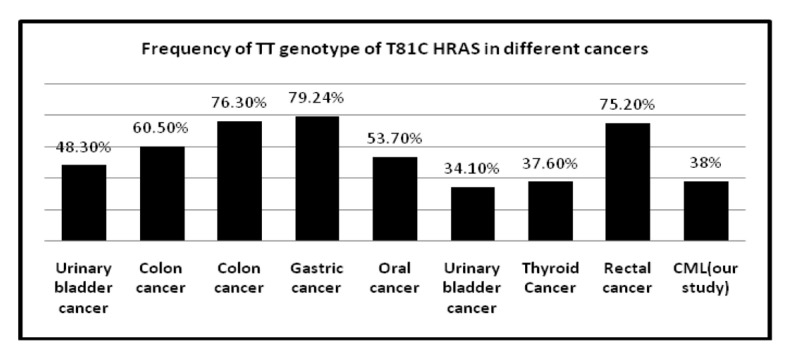
Frequency of TT genotype of T81C H-RAS in different cancers.

**Figure 4:**
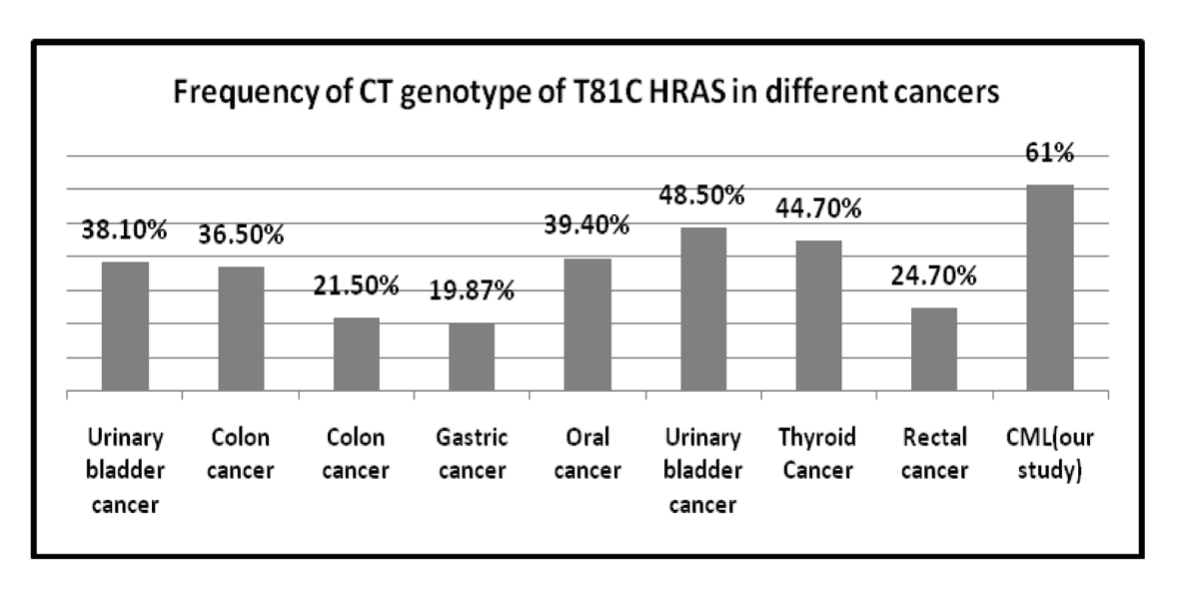
Frequency of CT genotype of T81C H-RAS in different cancers.

The frequency of T81C genotypes in different solid tumors is represented in Table 8 [14-16, 25, 29, 30] and also frequency of TT and CT genotype of T81C H-RAS in different cancers is shown Figures 3 and 4. Several molecular alternations in RAS gene have been identified such as minisatellites and mutations; however, research on the SNP in RAS gene was rare. This observation indicates that the distribution of H-RAS T81C polymorphism seems to be genetically different in various ethnics.

**Table 8:** Frequency of T81C Genotypes in Different Cancers

Cancer type	TT genotype	CT genotype	CC genotype	References
Rectal cancer	75.2%	24.7%	0%	16
Colon cancer	76.3%	21.5%	2.1%	16
Gastric cancer	79.24%	19.87%	0.89%	16
Thyroid cancer	37.6%	44.7%	17.7%	29
Urinary bladder cancer	48.3%	38.1%	13.4%	34
Colon cancer	60.5%	36.5%	3%	35
Oral cancer	53.7%	39.4%	6%	36
Urinary bladder cancer	34.1%	48.5%	17.4%	37

We found a strong association of H-RAS T81C polymorphism with the poor progression of CML. Compared to TT genotype, CT-genotype occurred more frequently in patients showing no molecular response and loss of hematogical response than good responders (P < 0.0001 and P < 0.003). Moreover, we found a statistically significant correlation of this polymorphism with thrombocytopenia with a higher of rare C allele in thrombocytoprenic group patients. We found a significant association of C allele occurrence in CML patients to the transition from CP CML to the BC (P < 0.0003).

### Conclusion

In conclusion, a frequent genetic polymorphism in the H-RAS proto-oncogene appears to be epidemiologically relevant for CML risk and progression. Its possible polymorphic loci in H-RAS could be a promising subject of additional studies.
